# Dry eye disease after LASIK

**Published:** 2012-03-05

**Authors:** L Ţuru, C Alexandrescu, D Stana, R Tudosescu

**Affiliations:** VISIONCLINIC, Bucharest; ’’Carol Davila’’ University of Medicine and Pharmacy, Bucharest; Scientific Resercher, Ophtalmology Clinic, University Emergency Hospital Bucharest

**Keywords:** LASIK, lacrimal film, dry eye disease, artificial tears

## Abstract

LASIK is a surgical tehnique for the correction of refractive errors (myopia, hyperopia, astygmatism). It results in a reshape of the cornea with ocular surface and especially tear film disease. It is a cause for a iatrogenic dry eye syndrome. Neurogenic and inflamatory theory explain this disease. The main therapy of dry eye is the replacement with artificial tears.

The refractive surgery became more important in the last years also in our country. This type of surgery aimes especially young people, fully active who want to improve their quality of life and through this procedure not to depend on glasses or contact lenses. One technique used in refractive surgery on the cornea is the LASIK procedure.

But any action on the body results in a reaction on its part, meaning that the „polishing” of the cornea will determine a local complex ocular response - the dry eye.

**The LASIK surgery **(laser in situ keratomileusis) represents the use of a special type of laser (excimer laser) under a corneal flap (in situ) in order to reshape the cornea (keratomileusis) [**1**]. The surgical procedure begins with the creation of a thin flap with a nasal or superior hinge using a microkeratome. After the reflection of the flap, the corneal tissue will be ablated with the excimer laser using a protocol specific for each pacient. The flap is repositioned and a contact lens can be used 24 hours postoperatively for protection. Through this technique refractive errors( myopia – 8 D, hyperopia +5 D, astigmatism 5 D) can be corrected depending on the thickness of the cornea(pachymetry) [**2**]. The pacients older than 21 years with a stable refraction (the same values of the diopters) in the last 2 years may benefit from this procedure.

This type of surgery influences the ocular surface and the tear film.

**The dry eye syndrome **is a multifactorial disease of the ocular surface caused by an inadequate quantity or quality of the tears [**3**]. The tear film has a defensive role (the protection of the ocular surface) and a refractive role (the first ocular diopter at the air – cornea surface). It has 3 layers: lipid, aqueous and mucuous. Any disturbance in the composition of one of the layers can lead to ocular dryness.

In order to have postoperative results satisfying for the physician and for the pacient, it is required to have a good selection of the cases which can benefit from this procedure and to perform tests and preoperative specific measurements.

• Slit-lamp examination of the ocular surface with the assesment of the lacrimal meniscus, LIPCOF(Lid Parallel Conjunctival Folds), a careful examination of the eyelid margin and of the meibomian glands [**4**]

• Schirmer test

• TFBUT (Tear Fluorescein Breakup Time)

• The corneal map using the corneal topography (the follow-up of the tear film in dynamics)

• Corneal sensibility Analysis (Ocular Response Analyzer - ORA) 

The ocular surface and the lacrimal gland work together as a functional unit for the production and distribution of the tears. The sensitive nerves from the corneal epithelium and stroma are the triggers for blinking through which the tears are spread on the ocular surface and after that the used tears are pumped in the nasolacrimal duct.

**The mechanism for the dry eye syndrome post LASIK **can be explained by 2 theories : neurogenic and inflamatory, chained in a vicious circle [**5**].

*The neurogenic theory *The surgical destruction (microkeratome, stromal ablation) of the nerve enndings from the subepithelial plexus produces hypoaestesia with a decrease in the stimulation of the lacrimal gland secretion, thus a aqueous lacrimal deficiency. Also a denervated cornea determines a drop in the blink rate. Thereby the ocular surface is exposed for a longer period of time increasing the evaporation of the tears. All of these lead to a low lacrimal clearance.

*The inflamatory theory *A corneal aggression, even iatrogenic (LASIK surgery) increases the production of pro-inflammatory citokines leading to the release of inflammatory mediators which will exacerbate the corneal nerves lesions. It is described a neurotrophic epitheliopathy with an incidence of approximately 4% between 1 and 3 months postoperatively [**6**]. Through confocal microscopy determinations, it was established that post LASIK the number of stromal nerve fibers decreases with 90%.

One year after the surgery , the measurements have reached 50% nerve fibers compared with the preoperative ones.

However there are studies showing corneal sensibility comeback at 6 months after the surgery by comparing preoperatory and postoperatory corneal histeresis (CH) measured with ORA(Ocular Response Analyser Reichert) [**7**].

Most of the pacients will develop ocular dryness in the first 3-6 months after the surgery, considered „transient”.

*Surgical risk factors *involved in the development of the postoperative dry eye syndrome are related to:

• Hinge (smaller width, superior position – sectioning of the nerve fibers from the nasal and temporal subepithelial plexus)

• Flap (a large diameter, thicker, done mechanically or with the femtosecond laser [**8**])

• Larger ablation ( for the pacients with higher diopters)

*Other risk factors *for the development of dry eye syndrome post LASIK are:

• Female sex

• Smoking

• Moderate or large refractive errors/ the size of the ablation

• Low corneal sensibility

• Diseases of the ocular surface(chronic seborrheic blepharitis)

• Preoperative dry eye symptoms subjectively described by the pacients

• Environmental factors(air conditioning, pollution, wind)

• The use of computers(long periods of watching the computer)

**The symptoms **vary from discreet ocular discomfort, foreign body sensation, burning sensation. They tend to apear towards the end of the day and are emphasized by the environmental factors, requiring the frequent use of ocular lubricants. What is typical is the need to blink more often in ordet to clarify the image, the pacients feeling a real discomfort impairing the quality of vision. 

**The treatment after LASIK surgery **consists of 2 types of eye drops adminstered topically: a set combination between an antibiotic and cortisone for 2 weeks and preservative – free artificial tears for minimum 1 month.

When the pacients suffer from **ocular dryness**, the treatment with lacrimal replacement therapy is extended even up to 1 year, avoiding formulas containing preservatives (cumulative toxic effect).

The perfect artificial tear does not exist because it does not have all the ingredients which can be found in the natural tears. There is not a type of product that can treat all the persons. The ideal formula should fulfill requirements such as:

• A composition similar to that of the natural tears(helps with tha healing process)

• No preservatives(minimal toxic effect) 

• To be stable on the ocular surface (fewer adminstrations)

• Not to cloud the vision (not to disrupt the daily activity)

The artificial tears [**9**] may contain:

• high viscosity agents

• electrolites

• solutions to maintain the ocular tonicity(NaCl, KCl)

• agents for adjusting pH, ideally 7.3

• agents which lenghten the retention time on the eye

• preservatives for maintaining the solution’s sterility (preferably without)

The preservatives are used to prevent the bacterial contamination of the eye drops, thus having sterile products. The most widely used is Benzalkonium Chloride BAK. The presevatives’ side effects on the ocular surface are visible on the tear film, conjunctiva and cornea. Because of their detergent properties, they dissolve the tear film’s lipids which leads to its instability, to increased evaporation, thus ocular dryness. On the conjunctiva, the preservatives cause the goblet cells’ destruction, altering the tear film’s composition. They induce the microvili destruction on the cornea and ruptures in the tight jonctions, thus the tearing of the epithelial barrier. This increases the cornea’s permeability, but it also slows the healing process. Therefore, the polytherapy often does more harm than good.

If the simptoms do not improve with lacrimal substitutes and their frequent adminstration becomes a discomfort, the lacrimal puncta may be temporarily occluded with devices called „plugs”. 

## Conclusions

The dry eye syndrome after LASIK surgery is a reality, a consequence of the direct damage on the ocular surface, with the disturbance on the tear film forming mechanisms. That is why it is necessary to have a detailed consultaltion, an accurate information of the patient who will undergo refractive surgery. Most of the pacients have ocular dryness in the first months after the surgery, but that can be managed through treatment and the education of the patient. But sometimes it can create a genuine ocular discomfort with a decrease in the quality of life, requiring a replacement therapy with artificial tears for a longer period of time (months, 1 year).
